# Disease-specific distress healthcare financing and catastrophic out-of-pocket expenditure for hospitalization in Bangladesh

**DOI:** 10.1186/s12939-022-01712-6

**Published:** 2022-08-20

**Authors:** Nurnabi Sheikh, Abdur Razzaque Sarker, Marufa Sultana, Rashidul Alam Mahumud, Sayem Ahmed, Mohammad Touhidul Islam, Susan Howick, Alec Morton

**Affiliations:** 1 Department of Management Science, Strathclyde Business School, University of Strathclyde, Glasgow, UK; 2 Population Studies Division, Bangladesh Institute of Development Studies (BIDS), Dhaka, Bangladesh; 3 Deakin Health Economics, Institute for Health Transformation, School of Health and Social Development, Deakin University, Geelong, Victoria-3220 Australia; 4 NHMRC Clinical Trials Centre, Faculty of Medicine and Health, The University of Sydney, Sydney, Australia; 5 Institute of Health and Wellbeing, Health Economics and Health Technology Assessment, University of Glasgow, Glasgow, UK; 6 World Health Organization Country Office for Bangladesh, Dhaka, Bangladesh

**Keywords:** Out-of-pocket payment, Catastrophic health expenditure, Distress financing, Universal Health Coverage, Inequalities, Bangladesh

## Abstract

**Background:**

Financial risk protection and equity are two fundamental components of the global commitment to achieve Universal Health Coverage (UHC), which mandates health system reform based on population needs, disease incidence, and economic burden to ensure that everyone has access to health services without any financial hardship. We estimated disease-specific incidences of catastrophic out-of-pocket health expenditure and distress financing to investigate progress toward UHC financial risk indicators and investigated inequalities in financial risk protection indicators by wealth quintiles. In addition, we explored the determinants of financial hardship indicators as a result of hospitalization costs.

**Methods:**

In order to conduct this research, data were extracted from the latest Bangladesh Household Income and Expenditure Survey (HIES), conducted by the Bangladesh Bureau of Statistics in 2016–2017. Financial hardship indicators in UHC were measured by catastrophic health expenditure and distress financing (sale/mortgage, borrowing, and family support). Concentration curves (CC) and indices (CI) were estimated to measure the pattern and severity of inequalities across socio-economic classes. Binary logistic regression models were used to assess the determinants of catastrophic health expenditure and distress financing.

**Results:**

We found that about 26% of households incurred catastrophic health expenditure (CHE) and 58% faced distress financing on hospitalization in Bangladesh. The highest incidence of CHE was for cancer (50%), followed by liver diseases (49.2%), and paralysis (43.6%). The financial hardship indicators in terms of CHE (CI = -0.109) and distress financing (CI = -0.087) were more concentrated among low-income households. Hospital admission to private health facilities, non-communicable diseases, and the presence of chronic patients in households significantly increases the likelihood of higher UHC financial hardship indicators.

**Conclusions:**

The study findings strongly suggest the need for national-level social health security schemes with a particular focus on low-income households, since we identified greater inequalities between low- and high-income households in UHC financial hardship indicators. Regulating the private sector and implementing subsidized healthcare programmes for diseases with high treatment costs, such as cancer, heart disease, liver disease, and kidney disease are also expected to be effective to protect households from financial hardship. Finally, in order to reduce reliance on OOPE, the government should consider increasing its allocations to the health sector.

## Background

In 2015, the United Nations declared the Sustainable Development Goals (SDGs); Universal Health Coverage (UHC) is one of the key targets of the proposed health-related SDGs [1]. The UHC target states that equitable health access should be available for all citizens at affordable costs without them facing any financial hardship [2]. The absence of financial risk protection in terms of equitable health access has made UHC difficult for many low-and middle-income countries (LMICs) [3]. Many countries, including Bangladesh, adopted UHC as a priority for their health systems to improve health access for citizens. However, in the absence of proper risk pooling mechanisms, the majority of citizens in LMICs experience high out-of-pocket expenditure (OOPE) on healthcare, putting them in financial hardship [4]. As a result, households are forced to borrow money, sell assets, or reach out for assistance from friends or relatives, and they often experience catastrophic health expenditure (CHE) [4].

About 150 million people globally experience CHE each year due to high OOP healthcare payments while 90% of the people who experienced CHE live in low resource countries [5]. In Bangladesh, total health expenditure (THE) in 2015 comprised 67% OOPE by households, followed by 23% public funding, with another major source of health financing being development partners [4]. About 5 million people in Bangladesh fall below the poverty line each year due to high OOP healthcare spending [6]**.** A study found that 14.2% of households in Bangladesh faced CHE at the 10% of total household consumption expenditure (THCE) threshold based on the national representative Household Income Expenditure Survey 2010 [6]**.** This financial burden of CHE increased to 24.7% of THCE between 2010 and 2016 [2]. Furthermore, according to a recent study in Bangladesh, about 43% of households who used healthcare did so by selling properties, borrowing, or receiving assistance from relatives [7]. Nevertheless, they do not investigate the factors that lead to such high levels of distress financing in that study. Only a few studies have investigated CHE and distress financing related to OOPE in Bangladesh [3, 6, 8–10].

Moreover, the severity of disease-specific financial burden owing to hospitalization is also rarely addressed in Bangladesh. Developed and developing countries are both experiencing burden from communicable and non-communicable diseases, although developing countries are suffering the most as a result of their demographic and socioeconomic transitions [11]. In Bangladesh, non-communicable diseases like cardiovascular diseases, cancer, chronic respiratory diseases, and diabetes are on the rise [12]. In addition, communicable diseases like malaria, tuberculosis, acute respiratory infection, and diarrheal diseases are also affecting Bangladesh with an increasing rate [13].

A number of studies in Bangladesh have assessed health expenditures for distinct diseases such as diarrhea, pneumonia, typhoid, and others [14–16]. Two studies have investigated disease-specific financial burden and its impact on households, but they were limited to specific geographical areas with limited sample sizes [8, 9] and thus do not provide a national representative estimate. However, to our knowledge, no studies in Bangladesh have studied disease-specific OOPE on hospitalization considering both communicable and non-communicable diseases as well as the impact on households, using a nationally representative survey. In compliance with international and national commitment to SDG-3, health system reform based on population needs, disease incidence, and economic burden is fundamental for the path to UHC. Countries are simultaneously planning health-system reforms based on disease burdens and population dynamics, but Bangladesh has paid little attention to this [9]. As a result, estimating disease-specific OOPE and its financial impact on households may provide policy-driven evidence to adopt reform policies.

Furthermore, seeking healthcare from private facilities was also cited as a significant obstacle since, due to high OOPE and low affordability, it could impose a substantial economic burden on households, particularly low-income households [17]. Nevertheless, studies on catastrophic OOPE do not provide healthcare providers’ differential estimates since these were limited to either public or private healthcare providers [8, 9, 14–16]. We believe that OOPE and its repercussions vary by disease and type of healthcare provider (public or private) in Bangladesh. Inequalities are another major concern for healthcare systems in low resource countries like Bangladesh, where socio-economically disadvantaged populations do not get proper access to healthcare due to their financial inability [18]. However, before adopting new policies to reduce inequalities, it is necessary to recognize which socio-economic groups have less access to healthcare and suffer most from the impact of high OOPE.

The aim of this study is to estimate disease-specific incidence of distress financing and catastrophic OOPE. This will help to assess progress toward UHC financial risk indicators based on hospitalization in public and private facilities in Bangladesh and will also investigate inequalities in financial risk protection indicators through wealth quintiles. In addition, the study explores the determinants of financial hardship indicators as a result of hospitalization costs. Disease and provider-specified estimations are expected to aid policymakers in developing evidence-based policies on alternative financial mechanisms to safeguard households, allowing them to contribute more to UHC. This will include providing policymakers with an understanding of which diseases should be targeted for coverage under the government’s first social health protection scheme, locally known as ‘Shastho Surokhsma Karmasuchi’ (a pilot-based healthcare financing scheme for the pro-poor population) [19].

## Methods

### Data sources

This analysis utilized data from the latest Bangladesh Household Income and Expenditure Survey (HIES), conducted by the Bangladesh Bureau of Statistics (BBS) in 2016–2017 [20]. This is a nationally representative survey which followed a two-stage stratified random sampling technique. At the first stage, a total of 2,304 primary sampling units (PSUs) were randomly selected from each of the administrative areas according to probability proportional to size (PPS) sampling. In the second stage, 20 households from each of the selected PSUs were randomly selected from the prior household lists. Finally, information on income, expenditure and consumption including health expenditures were collected from 46,080 households by trained data collectors. A total of 45,423 household’s (31,621 from rural and 13,802 from urban areas) data were included in this analysis after excluding missing and anomalous information from the main data set.

Respondents were requested to provide information relating to hospitalization (defined as an overnight stay) for 365 days preceding to the survey. Data on the place of hospitalization, reasons (diseases) for hospitalization, expenditures, and mode of financing the cost of treatment were also collected followed by hospitalization incidence. Expenditure on hospitalization covers both medical and non-medical expenses. The medical expenses comprised operation cost, consultation/doctor fees, bed/cabin charges, medicines, and medical investigations. The non-medical expenses included transport cost, informal tips, and other formal charges. The OOPE considered both medical and non-medical expenses on hospitalization in this study. In our analysis, we included 22 diseases or reasons for hospitalization such as diarrhea/dysentery, fever, pain, injury/accident, blood pressure, heart disease, cancer, diabetes, etc., as per data recorded in HIES 2016 survey and we then classified these into communicable and non-communicable. In our analysis communicable diseases include diarrhea/dysentery, pneumonia, malaria, jaundice, skin diseases etc., whereas, non-communicable diseases (NCDs) include blood pressure, heart diseases, respiratory diseases/asthma, cancer, kidney diseases, liver diseases, etc. We considered injury/accident, fever, pain and mental health diseases as NCDs [21]. Hospital facilities were likewise divided into two categories: public and private.

### Outcome measures

#### Incidence of catastrophic health expenditure

Two established definitions are frequently used for the estimation of CHE. One divides OOP healthcare expenditure by total household consumption expenditure and the other divides OOP healthcare expenditure by total non-food consumption expenditure (NFE) [6, 22, 23]. In this study, we used both of the definitions to estimate CHE for hospitalization in Bangladesh. However, there is no single recognized threshold to consider CHE estimation. In this analysis, a 10% threshold of THCE and a 25% threshold of NFE were used to determine CHE in Bangladesh. If a household’s OOPE were more than 10% of the THCE and more than 25% of NFE, then this was measured as a CHE incidence for that household. Two dummy variables were created for each of the two thresholds and recoded “yes” if household healthcare expenditure was more than the threshold and “no” otherwise. OOPE is defined as the total of all medical and non-medical direct payments made by households/patients to purchase inpatient healthcare services [4]. In addition, food expenditure data was taken from HIES 2016, which collected data on daily and weekly consumption and classified it into 17 food bundles including food grains, pulses, fish, egg, meat, vegetables, etc. Similarly, non-food expenses were divided into 21 categories in HIES such as fuel and lighting, cosmetics, washing and cleaning, education, transport, cloths, footwear etc., and collected based on monthly and yearly consumption. However, since we computed medical expenses separately, we omitted them from non-food expenditure in our analysis. The incidence of CHE was estimated for each of the 22 diseases/reasons for hospitalization, including for communicable and non-communicable diseases.

#### Distress health financing for financial difficulties

Another outcome variable is distress health financing due to OOPE on hospitalization. Distress financing defines funding for OOPE by selling or mortgaging household assets/lands, borrowing money from lender/banks/friends/relatives, and by receiving assistance from friends/relatives. If a household incurred OOPE and managed money from any of these sources then a dummy variable was coded “yes” as a measure of distress financing, and “no” otherwise. The incidence of distress financing was also calculated for each of the 22 diseases/reasons for hospitalization, as well as communicable and non-communicable diseases.

### Explanatory variables

The number of children aged under-five years old, number of female members, number of elderly members (65 years and above), number of members that earn a wage, number of members suffering from chronic illness, number of members hospitalized in the last 12 months, household size, place of residence and wealth quintile were considered as predictor variables in this analysis. These predictors were chosen based on a review of previously published literature [3, 6, 10, 24–26]. The wealth quintile was generated by considering a list of household assets. Scores for each of the households were generated for household assets by using principal component analysis (PCA) and the scores were categorized into five equal parts from the lowest to highest 20%.

### Statistical analysis

This study used descriptive statistics, concentration curve, concentration index, and logistic regression analysis. Proportion, mean, and standard deviation were used to present descriptive data. Concentration curves and indices for the incidence of CHE and distress financing were created for equality analysis. These examine the pattern and severity of inequalities across socio-economic classes measured by asset quintiles. The computational formula for the concentration index is as follows:

$$\mathrm{CI}=\frac2{\mathrm n^2\overline{\mathrm y}}{\textstyle\sum_{\mathrm i=1}^{\mathrm n}}{\mathrm y}_{\mathrm i}\;{\mathrm r}_{\mathrm i}$$

where CI is the concentration index which lies between -1 and + 1; $\overline{y }$ is the mean of outcome measures (i.e., hospitalization, CHE and distress financing); n is the number of individuals; and ${r}_{i}$ is the cumulative rank proportion of the individual according to wealth index. The CI will be negative when concentration curve lies above the equity line which means the outcome is more concentrated among the poor. On the other hand, when the concentration curve lies below the equality line, the concentration index will be positive, which indicates outcome is concentrated more among the rich. The predictors of CHE and distress financing owing to OOPE on hospitalization were investigated using binary logistic regression models, with the results provided as odds ratios (i.e., exponential form of regression coefficient, OR = exp (beta)) and 95% confidence intervals. The regression model can be expressed as-

$$\mathrm{logit }(\mathrm{Yi})\hspace{0.17em}=\hspace{0.17em}\mathrm{\alpha }\hspace{0.17em}+\hspace{0.17em}{\upbeta }_{1}{\mathrm{X}}_{1\mathrm{i}}\hspace{0.17em}+\hspace{0.17em}{\upbeta }_{2} {\mathrm{X}}_{2\mathrm{i}}\hspace{0.17em}+\hspace{0.17em}\dots \dots \dots \hspace{0.17em}+\hspace{0.17em}{\upepsilon }_{\mathrm{i}}$$

Where Y_i_ is the dichotomous outcome variables (i.e., CHE and distress financing) with value 0 if household did not experience CHE and distress financing and 1 if household faced CHE and experience distress financing; α is the constant; β_1_, β_2_…. are the regression coefficients for the corresponding explanatory variables; X_1i_, X_2i_…..denote explanatory variables; and ϵ_i_ is the error term. To build the regression model, we first identified significant explanatory variables from the published literature and explored bivariate relationships between variables. Explanatory variables that were found statistically significant during the bivariate analysis were included in our regression models. In our analysis, we investigated individual-level data to estimate disease-specific hospitalization incidence, length of hospital stays, and disease-specific OOPE, as well as household-level data to estimate CHE and distress healthcare financing.

## Results

This study looked at the data of 183,757 individuals from 45,423 households (Table 1). Among the total households, more than one-third had one or more children under the age of five, 18.1% had at least one elder member, and the majority had at least one earning member. Almost one-third of households had one member who had suffered from chronic illness/disability in the 12 months preceding the survey. The majority of the households were from rural rather than urban areas (70% vs. 30%) and were almost equally distributed among the socio-economic quintiles.

**Table 1:** Background and household characteristics of the study participants

Variables	n	%
**Individual participants characteristics (n = 183,757)**		
** Gender of the participants**		
*Male*	91,414	49.8
*Female*	92,343	50.3
** Age of the participants**		
*Children (0–14 yrs.)*	58,298	31.7
*Working age (15–64 yrs.)*	115,883	63.1
*Elderly (65 yrs. and above)*	9,576	5.2
**Number of Households**	45,423	
**Number of individuals**	183,757	
**Number of hospitalizations**	3,220	
**Household characteristics (n = 45,423)**		
**Number of female members**		
*None*	310	0.7
*One*	15,383	33.9
*2 and more*	29,730	65.5
**Number of U5 children**		
*None*	27,820	61.3
*One*	14,201	31.3
*2 and more*	3,402	7.5
**Number of elder members (65 years & above)**		
*None*	37,192	81.9
*One*	6,925	15.3
*2 and more*	1,306	2.9
**Number of earning members**		
*One*	34,098	75.1
*Two*	8,802	19.4
*3 and more*	2,523	5.6
**Number of members suffering from chronic illness/disability in last 12 months**		
*None*	23,423	51.6
*One*	13,357	29.4
*Two*	6,676	14.7
*3 and more*	1,967	4.3
**Number of members hospitalized in last 12 months**		
*None*	42,410	93.4
*One*	2,837	6.3
*2 and more*	176	0.4
**Household size**		
*1–2*	6,288	13.8
*3–4*	23,877	52.6
*5 and above*	15,258	33.6
**Mean household size (Mean ± SD)**	4.1 ± 1.6	
**Administrative division**		
*Barishal*	4,234	9.3
*Chattogram*	7,801	17.2
*Dhaka*	9,223	20.3
*Khulna*	7,135	15.7
*Mymensingh*	2,849	6.3
*Rajshahi*	5,642	12.4
*Rangpur*	5,685	12.5
*Sylhet*	2,854	6.3
**Place of residence**		
*Rural*	31,621	69.6
*Urban*	13,802	30.4
**Wealth quintiles**		
*Lowest 20%*	9,332	20.5
*2nd*	8,824	19.4
*3rd*	9,069	20.0
*4th*	9,203	20.3
*Upper 20%*	8,995	19.8

### Incidence of diseases-specific hospitalization rate and length of hospital stay

Table 2 shows the hospitalization rate and length of hospital stay for various diseases in Bangladesh. In the 365 days before to the survey, the overall hospitalization rate was 3220 per 183,757 population (i.e., 175 per 10,000 population). The overall hospitalization rate was higher in public health facilities (approximately 59%) and due to non-communicable diseases (approximately 53%). Child delivery and pregnancy-related diseases (17.5%) were the most common reason for hospitalization among the 22 diseases/reasons studied, followed by injury/accident (11.5%) and diarrhea/dysentery (10.9%).

**Table 2:** Health care provider specific-hospitalization (12 months prior to the survey) and average length of hospital stay by diseases in Bangladesh

Diseases/reasons for hospitalization	Hospitalization in last 12 months n (%)			Length of hospital stay (in days) (Mean ± SD)		
	**Health care provider**			**Health care provider**		
	**Public**^**b**^	**Private**^**b**^	**All**^**a**^	**Public**	**Private**	**All**
Diarrhea/Dysentery	288 (82.1)	63 (17.9)	351 (10.9)	4.0 ± 4.7	2.9 ± 1.7	3.8 ± 4.3
Fever	142 (75.5)	46 (24.5)	188 (5.8)	4.8 ± 3.9	5.3 ± 3.4	4.9 ± 3.8
Pain	171 (63.6)	98 (36.4)	269 (8.4)	9.0 ± 25.2	5.7 ± 10.3	7.8 ± 21.0
Injury/Accident	260 (70.5)	109 (29.5)	369 (11.5)	9.1 ± 11.6	12.3 ± 20.3	10.1 ± 14.7
Blood pressure	78 (70.9)	32 (29.1)	110 (3.4)	5.7 ± 7.5	4.5 ± 2.8	5.4 ± 6.5
Heart disease	145 (63.9)	82 (36.1)	227 (7.1)	8.0 ± 15.6	9.0 ± 7.9	8.4 ± 13.3
Respiratory Diseases/ Asthma/Bronchitis	146 (69.2)	65 (30.8)	211 (6.6)	6.9 ± 7.2	6.1 ± 5.5	6.6 ± 6.7
Weakness/Dizziness	64 (64.0)	36 (36.0)	100 (3.1)	4.7 ± 5.5	4.4 ± 7.3	4.6 ± 6.2
Pneumonia	96 (73.3)	35 (26.7)	131 (4.1)	5.5 ± 3.5	6.9 ± 5.3	5.8 ± 4.1
Tuberculosis	8 (57.1)	6 (42.9)	14 (0.4)	20.1 ± 20.3	19.8 ± 39.3	20.0 ± 28.6
Malaria	6 (66.7)	3 (33.3)	9 (0.3)	7.5 ± 7.1	3.7 ± 2.9	6.2 ± 6.1
Jaundice	18 (62.1)	11 (37.9)	29 (0.9)	6.6 ± 7.0	5.8 ± 5.6	6.3 ± 6.4
Female diseases	52 (36.1)	92 (63.9)	144 (4.5)	7.4 ± 9.1	7.2 ± 6.4	7.3 ± 7.4
Child delivery and pregnancy-related diseases	169 (30.0)	395 (70.0)	564 (17.5)	5.5 ± 4.4	5.5 ± 2.7	5.5 ± 3.3
Cancer	25 (52.1)	23 (47.9)	48 (1.5)	28.1 ± 44.1	23.2 ± 37.6	25.7 ± 40.7
Mental health	22 (78.6)	6 (21.4)	28 (0.9)	12.3 ± 13.3	19.3 ± 17.1	13.8 ± 14.2
Paralysis	43 (69.4)	19 (30.6)	62 (1.9)	7.6 ± 7.0	11.2 ± 9.4	8.7 ± 7.9
Scabies/Skin diseases	19 (67.9)	9 (32.1)	28 (0.9)	10.2 ± 13.8	7.8 ± 6.4	9.4 ± 11.9
Kidney Diseases	54 (47.8)	59 (52.2)	113 (3.5)	12.7 ± 17.7	9.4 ± 9.6	11.0 ± 14.1
Liver Diseases	35 (55.6)	28 (44.4)	63 (2.0)	11.0 ± 13.1	8.4 ± 8.6	9.8 ± 11.4
Ear/ ENT problems	15 (46.9)	17 (53.1)	32 (1.0)	11.7 ± 18.3	6.1 ± 4.7	8.7 ± 13.1
Eye problem	40 (30.8)	90 (69.2)	130 (4.0)	5.3 ± 4.9	4.9 ± 6.6	5.0 ± 6.1
**All diseases**	**1,896 (58.9)**	**1,324 (41.1)**	**3,220 (100.0)**	**7.3 ± 13.0**	**7.0 ± 10.3**	**7.2 ± 12.0**
Communicable	841 (55.3)	679 (44.7)	1,520 (47.2)	5.4 ± 6.0	5.8 ± 5.5	5.6 ± 5.8
Non-communicable	1,055 (62.1)	645 (37.9)	1,700 (52.8)	8.8 ± 16.4	8.2 ± 13.6	8.6 ± 15.4

^a^ Percentages based on column total

^b^ Percentages based on row total

The average length of hospital stay in Bangladesh was 7.2 days (SD = 12); a similar pattern was observed for both public (7.3 days with SD = 13) and private (7 days with SD = 10.3) hospitals, whereas, non-communicable diseases contributed a larger proportion of hospital stays (8.6 days with SD = 15.4) as compared to communicable diseases (5.6 days with SD = 5.8). The longest length of hospital stay was due to cancer (25.7 days with SD = 40.7), followed by tuberculosis (20 days with SD = 28.6), mental health (13.8 days with SD = 14.2), and injury/accident (10.1 days with SD = 14.7).

### Diseases-specific out-of-pocket expenditure on hospitalization

**Fig. 1:**
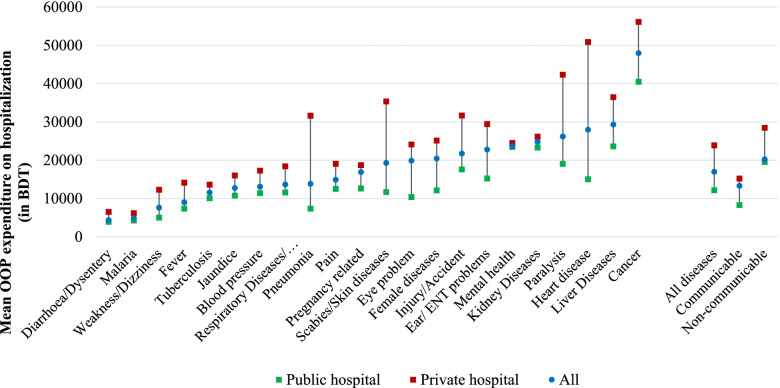
Mean OOP expenditure on hospitalization according to diseases/reasons for hospitalization and hospital types in Bangladesh

The average disease-specific OOPE on hospitalization by healthcare providers is presented in Fig. 1. The average yearly OOPE on hospitalization for all diseases was BDT 16,985 (SD = 29,444) (Table 3). This study reveals that the average OOPE on hospitalization was almost twice as high in private hospitals (BDT 23,899 with SD = 36,864) than public hospitals (BDT 12,156 with SD = 21,614). At the same time, non-communicable diseases had 1.5 times higher OOPE (BDT 20,247 with SD = 34,303) as compared with communicable diseases (BDT 13,336 with SD = 22,266). The highest OOPE was reported for cancer (BDT 47,983 with SD = 47,735), in both public (BDT 40,488 with SD = 35,630) and private hospitals (BDT 56,131 with SD = 57,878), followed by liver diseases (BDT 29,328 with SD = 30,143), and heart diseases (BDT 27,983 with SD = 52,140) respectively.

**Table 3:** Health care provider specific mean out-of-pocket healthcare expenditure on hospitalization by diseases in Bangladesh

Diseases/reasons for hospitalization	OOP expenditure (in BDT) due to hospitalization in last 12 months		
	**Health care provider**		
	**Public**	**Private**	**All**
	**Mean ± SD**	**Mean ± SD**	**Mean ± SD**
Diarrhea/Dysentery	3911 ± 4456	6501 ± 4599	4375 ± 4585
Fever	7384 ± 12,529	14,146 ± 13,511	9039 ± 13,068
Pain	12,522 ± 47,866	19,074 ± 41,124	14,909 ± 45,557
Injury/Accident	17,604 ± 23,268	31,687 ± 30,060	21,764 ± 26,222
Blood pressure	11,393 ± 11,658	17,295 ± 17,157	13,110 ± 13,674
Heart disease	15,037 ± 15,751	50,875 ± 79,458	27,983 ± 52,140
Respiratory Diseases/ Asthma/Bronchitis	11,582 ± 17,554	18,400 ± 15,969	13,682 ± 17,333
Weakness/Dizziness	5014 ± 5169	12,306 ± 17,488	7639 ± 11,726
Pneumonia	7364 ± 8310	31,639 ± 99,655	13,850 ± 52,575
Tuberculosis	10,069 ± 9123	13,617 ± 14,003	11,589 ± 11,115
Malaria	4267 ± 2674	6200 ± 3811	4911 ± 3005
Jaundice	10,776 ± 10,213	16,040 ± 14,370	12,772 ± 11,993
Female diseases	12,146 ± 10,573	25,149 ± 19,268	20,454 ± 17,760
Child delivery and pregnancy-related diseases	12,632 ± 12,526	18,735 ± 19,043	16,906 ± 17,562
Cancer	40,488 ± 35,630	56,131 ± 57,878	47,983 ± 47,735
Mental health	23,467 ± 30,268	24,517 ± 17,707	23,692 ± 27,764
Paralysis	19,068 ± 15,778	42,328 ± 47,969	26,196 ± 31,101
Scabies/Skin diseases	11,689 ± 11,622	35,378 ± 29,571	19,304 ± 21,819
Kidney Diseases	23,326 ± 22,836	26,168 ± 24,188	24,810 ± 23,490
Liver Diseases	23,613 ± 21,736	36,472 ± 37,366	29,328 ± 30,143
Ear/ ENT problems	15,239 ± 26,091	29,450 ± 21,045	22,789 ± 24,247
Eye problem	10,393 ± 10,069	24,112 ± 34,730	19,891 ± 30,054
**All diseases**	**12,156 ± 21,614**	**23,899 ± 36,864**	**16,985 ± 29,444**
Communicable	8312 ± 10,738	15,221 ± 26,959	13,336 ± 22,266
Non-communicable	19,560 ± 29,962	28,467 ± 42,495	20,247 ± 34,303

### Financial hardship indicators on hospitalization

**Fig. 2:**
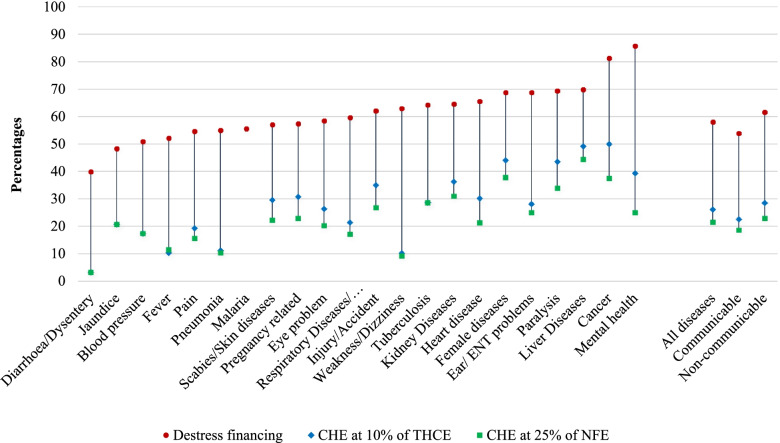
Incidence of distress and catastrophic health expenditure at 10% of THCE and 25% of NFE on hospitalization in Bangladesh

Figure 2 shows diseases-specific incidences of catastrophic health expenditure at 10% of THCE and at 25% of NFE and incidences of distress financing for financial hardship due to hospitalization.

Overall, 26.1% and 21.5% of households who had experienced at least one hospitalization in the 365 days prior to the survey, incurred CHE at 10% of THCE and at 25% of NFE respectively (Table 4). When household member(s) were hospitalized at private hospitals (36.1% at 10% of THCE and 28% at 25% of NFE) a household experienced a higher level of CHE compared with being hospitalized at a public hospital (19% at 10% of THCE and 16.9% at 25% of NFE). At the same time, hospitalization due to non-communicable diseases (28.5% at 10% of THCE and 22.9% at 25% of NFE) incurred a higher CHE as compared to communicable diseases (22.6% at 10% of THCE and 18.6% at 25% of NFE). A similar pattern occurs when looking at either public or private hospitals i.e., CHE was comparatively higher among those who were treated at private rather than public hospitals. The highest incidence of CHE was for cancer (50%), followed by liver diseases (49.2%), and paralysis (43.6%) at 10% of THCE.

**Table 4:** Health care provider specific incidence of catastrophic health expenditure and distress financing due to out-of-pocket spending on hospitalization in Bangladesh

Diseases/reasons for hospitalization	Catastrophic health expenditure due to hospitalization n (%)						Distress financing due to hospitalization n (%)		
	**10% of THCE**			**25% of NFE**			**Health care provider**		
	**Health care provider**			**Health care provider**					
	**Public**	**Private**	**All**	**Public**	**Private**	**All**	**Public**	**Private**	**All**
Diarrhea/Dysentery	9 (3.4)	2 (3.0)	11 (3.3)	7 (2.6)	4 (6.0)	11 (3.3)	120 (41.7)	20 (31.7)	140 (39.9)
Fever	11 (8.1)	8 (17.4)	19 (10.4)	15 (11.1)	6 (12.8)	21 (11.5)	73 (51.4)	25 (54.3)	98 (52.1)
Pain	24 (14.5)	23 (29.5)	47 (19.3)	22 (13.5)	16 (19.8)	38 (15.6)	81 (47.4)	66 (67.3)	147 (54.6)
Injury/Accident	72 (29.2)	52 (48.6)	124 (35.0)	57 (23.2)	38 (35.2)	95 (26.8)	158 (60.8)	71 (65.1)	229 (62.1)
Blood pressure	12 (16.0)	7 (20.6)	19 (17.43)	13 (16.9)	6 (18.8)	19 (17.4)	38 (48.7)	18 (56.3)	56 (50.9)
Heart disease	24 (16.7)	44 (53.3)	68 (30.2)	19 (13.4)	29 (34.9)	48 (21.3)	90 (62.1)	59 (72.0)	149 (65.6)
Respiratory Diseases/ Asthma/Bronchitis	25 (17.2)	20 (30.8)	45 (21.4)	18 (12.6)	18 (26.9)	36 (17.1)	87 (59.6)	39 (60.0)	126 (59.7)
Weakness/Dizziness	6 (9.7)	4 (11.4)	10 (10.3)	5 (8.2)	4 (11.1)	9 (9.3)	40 (62.5)	23 (63.9)	63 (63.0)
Pneumonia	6 (6.9)	8 (23.5)	14 (11.2)	8 (8.9)	5 (14.3)	13 (10.4)	49 (51.0)	23 (65.7)	72 (55.0)
Tuberculosis	3 (37.5)	1 (16.7)	4 (28.6)	3 (37.5)	1 (16.7)	4 (28.6)	5 (62.5)	4 (66.7)	9 (64.3)
Malaria	-	-	-	-	-	-	3 (50.0)	2 (66.7)	5 (55.6)
Jaundice	4 (22.2)	2 (18.2)	6 (20.7)	6 (33.3)	-	6 (20.7)	10 (55.6)	4 (36.4)	14 (48.3)
Female diseases	13 (25.5)	50 (54.4)	63 (44.1)	13 (24.5)	41 (45.6)	54 (37.8)	29 (55.8)	70 (76.1)	99 (68.8)
Child delivery and pregnancy-related diseases	27 (20.9)	134 (35.5)	171 (30.8)	28 (15.6)	99 (26.3)	127 (22.9)	106 (62.7)	218 (55.2)	324 (57.4)
Cancer	11 (44.0)	13 (56.5)	24 (50.0)	8 (32.0)	10 (43.5)	18 (37.5)	19 (76.0)	20 (87.0)	39 (81.3)
Mental health	8 (36.4)	3 (50.0)	11 (39.3)	5 (22.7)	2 (33.3)	7 (25.0)	19 (86.4)	5 (83.3)	24 (85.7)
Paralysis	17 (39.5)	10 (52.6)	27 (43.6)	14 (31.8)	7 (38.9)	21 (33.9)	29 (67.4)	14 (73.7)	43 (69.4)
Scabies/Skin diseases	4 (22.2)	4 (44.4)	8 (29.6)	4 (22.2)	2 (22.2)	6 (22.2)	11 (57.9)	5 (55.6)	16 (57.1)
Kidney Diseases	21 (38.2)	20 (34.5)	41 (36.3)	18 (33.3)	17 (28.8)	35 (31.0)	36 (66.7)	37 (62.7)	73 (64.6)
Liver Diseases	19 (54.3)	12 (42.9)	31 (49.2)	18 (51.4)	10 (35.7)	28 (44.4)	25 (71.4)	19 (67.9)	44 (69.8)
Ear/ ENT problems	3 (20.0)	6 (35.3)	9 (28.1)	2 (13.3)	6 (35.3)	8 (25.0)	9 (60.0)	13 (76.5)	22 (68.8)
Eye problem	7 (17.1)	27 (30.7)	34 (26.4)	5 (12.5)	21 (23.6)	26 (20.2)	25 (62.5)	51 (56.7)	76 (58.5)
**All diseases**	**336 (19.0)**	**451 (36.1)**	**787 (26.1)**	**298 (16.9)**	**349 (28.0)**	**647 (21.5)**	**1,062 (56.0)**	**806 (60.9)**	**1,868 (58.0)**
Communicable	108 (13.6)	218 (33.6)	326 (22.6)	100 (12.6)	168 (25.9)	268 (18.6)	435 (51.7)	385 (56.7)	820 (53.9)
Non-communicable	230 (22.9)	231 (37.6)	461 (28.5)	192 (19.1)	178 (28.9)	370 (22.9)	627 (59.4)	421 (65.3)	1,048 (61.6)

Numerator: number of households experienced CHE and distress financing, Denominator: number of households experienced at least one hospitalization for the specific diseases/reasons

On the other hand, households reported distress financing due to financial crisis for more than half (58%) of the total 3220 hospitalization cases and the intensity is higher among households who sought care from private hospitals (60.9%) as compared to public hospitals (56.0%). Likewise, this incidence was higher among those who were admitted due to non-communicable diseases (61.6%) as compared with communicable diseases (53.9%).

**Fig. 3:**
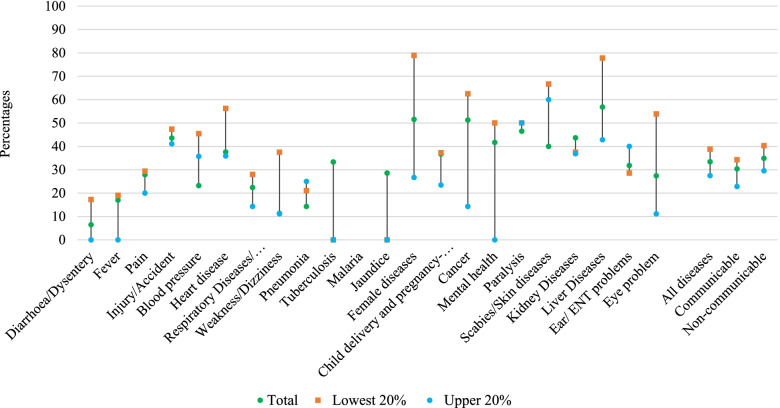
Disease specific-destress financing and catastrophic health expenditure at 10% of THCE on hospitalization in Bangladesh

Figure 3 depicts the diseases-specific incidence of both distress financing and CHE at 10% of THCE on hospitalization in Bangladesh. Thirty-three percent of households with at least one hospitalized case had to deal with both CHE and distress financing; this intensity is higher for non-communicable diseases (35%) as compared to communicable diseases (30%).

### Equity in financial hardship indicators

**Fig. 4:**
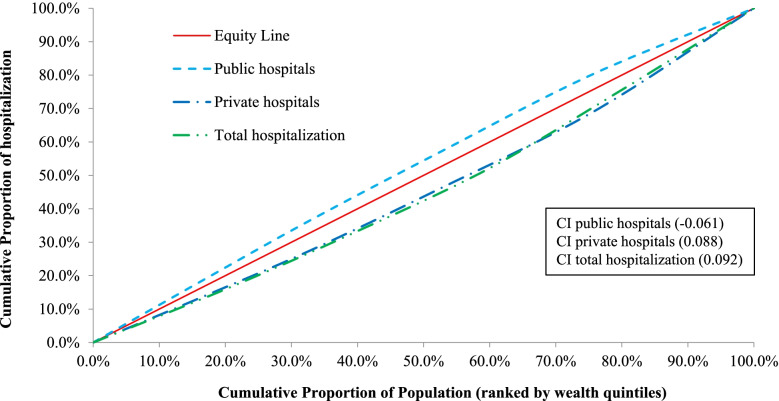
Concentration curves and indices for healthcare utilization from the public and private hospitals due to hospitalization

**Fig. 5:**
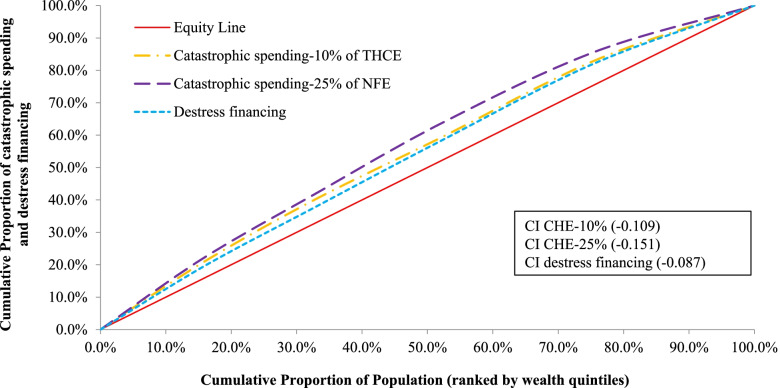
Concentration curves and indices for catastrophic spending and destress financing on hospitalization due to OOP healthcare spending

Figure 4 presents the concentration curves and indices to measure the inequality in access to healthcare on hospitalization among the socio-economic classes. The overall inpatient (hospitalization) healthcare utilization was more concentrated among the rich households compared to poor ones, while the poor have slightly higher access to the public hospitals. Moreover, private hospitals are used more by rich households. This shows that the poor have less access than the rich, and have to rely more on public facilities rather than private ones. Additionally, Fig. 5 illustrates concentration curves and indices for CHE and distress financing on hospitalization. All of the curves are over the equity line and negative concentration indices indicate that low-income households are more concentrated on both financial hardship indicators i.e., they experienced more CHE and distress financing compared with rich households. Furthermore, we identified that low-income households in Bangladesh had proportionally greater financial hardship than wealthy households for the majority of diseases, irrespective of communicable and non-communicable diseases (Fig. 3).

### Determinants of financial hardship

Table 5 indicates that statistically significant determinants of CHE due to the OOP health expenditure on hospitalization are the number of children aged under 5, number of members earning a wage, number of members suffering from chronic illness/disability, number of members hospitalized in last 12 months, type of hospital providers, diseases type, household size, administrative division, place of residence, and wealth quintiles.

**Table 5:** Determinants of catastrophic health expenditure and distress financing due to out-of-pocket health expenditure on hospitalization in Bangladesh

Variables	Catastrophic healthcare spending		Model C (Distress financing)
	**Model A (10% THCE)**	**Model B (25% NFE)**	
	**AOR (95% CI)**	**AOR (95% CI)**	**AOR (95% CI)**
**Number of female members**			
< *2 (ref)*	1.00	1.00	1.00
≥ *2*	0.92 (0.83, 1.02)	0.95 (0.84, 1.06)	0.93 (0.84, 1.03)
**Number of U5 children**			
*None (ref)*	1.00	1.00	1.00
*One*	1.00 (0.91, 1.10)	1.05 (0.94, 1.16)	1.02 (0.94, 1.11)
*2 and more*	1.03 (0.90, 1.18)	1.18^a^ (1.02, 1.36)	1.10 (0.98, 1.24)
**Number of elder members (≥ 65 years)**			
*None (ref)*	1.00	1.00	1.00
*One*	0.99 (0.88, 1.10)	0.95 (0.84, 1.06)	0.96 (0.85, 1.07)
*2 and more*	0.84 (0.68, 1.04)	0.97 (0.78, 1.20)	0.87 (0.73, 1.04)
**Number of earning members**			
*One (ref)*	1.00	1.00	1.00
*Two*	0.81^b^ (0.73, 0.90)	0.82^b^ (0.73, 0.92)	0.88^b^ (0.80, 0.96)
*3 and more*	0.69^b^ (0.59, 0.81)	0.56^b^ (0.47, 0.67)	0.82^b^ (0.73, 0.93)
**Number of chronic illness/disabilities in last 12 months**			
*None (ref)*	1.00	1.00	1.00
*One*	1.17^b^ (1.05, 1.31)	1.11 (0.98, 1.25)	1.40^b^ (1.27, 1.54)
*Two and more*	0.87 (0.77, 1.01)	0.89 (0.79, 1.03)	1.51^b^ (1.37, 1.67)
**Number of members hospitalized in last 12 months**			
*One (ref)*	1.00	1.00	1.00
*2 and more*	2.49^b^ (2.14, 2.90)	2.88^b^ (2.47, 3.37)	0.73^b^ (0.64, 0.84)
**Health care provider**			
*Public (ref)*	1.00	1.00	1.00
*Private*	3.03^b^ (2.78, 3.31)	2.28^b^ (2.08, 2.49)	1.37^b^ (1.27, 1.48)
**Diseases**			
*Communicable (ref)*	1.00	1.00	1.00
*Noncommunicable*	1.64^b^ (1.49, 1.79)	1.52^b^ (1.38, 1.67)	1.39^b^ (1.28, 1.50)
**Household size**			
*1–2 (ref)*	1.00	1.00	1.00
*3–4*	0.85 (0.69, 1.06)	0.75^a^ (0.60, 0.94)	0.88 (0.71, 1.08)
*5 and above*	0.67^b^ (0.53, 0.85)	0.62^b^ (0.48, 0.78)	1.00 (0.80, 1.25)
**Administrative division**			
*Barishal (ref)*	1.00	1.00	1.00
*Chattogram*	0.38^b^ (0.33, 0.44)	0.48^b^ (0.41, 0.55)	1.35^b^ (1.18, 1.53)
*Dhaka*	0.54^b^ (0.47, 0.63)	0.62^b^ (0.53, 0.73)	0.69^b^ (0.61, 0.79)
*Khulna*	0.53^b^ (0.46, 0.62)	0.67^b^ (0.57, 0.79)	1.36^b^ (1.19, 1.56)
*Mymensingh*	0.49^b^ (0.39, 0.62)	0.71^b^ (0.56, 0.89)	0.91 (0.74, 1.11)
*Rajshahi*	0.51^b^ (0.43, 0.60)	0.55^b^ (0.46, 0.65)	0.78^b^ (0.67, 0.90)
*Rangpur*	0.58^b^ (0.49, 0.69)	0.72^b^ (0.60, 0.86)	1.43^b^ (1.21, 1.67)
*Sylhet*	0.48^b^ (0.40, 0.58)	0.69^b^ (0.57, 0.84)	1.08 (0.92, 1.27)
**Place of residence**			
*Rural (ref)*	1.00	1.00	1.00
*Urban*	0.86^b^ (0.78, 0.94)	0.75^b^ (0.68, 0.84)	0.74^b^ (0.68, 0.80)
**Wealth quintile**			
*Lowest 20%*	2.78^b^ (2.39, 3.22)	2.98^b^ (2.53, 3.50)	3.56^b^ (3.12, 4.05)
*2nd*	2.49^b^ (2.15, 2.88)	2.60^b^ (2.21, 3.05)	2.11^b^ (1.88, 2.38)
*3rd*	2.01^b^ (1.75, 2.31)	2.64^b^ (2.27, 3.08)	2.23^b^ (2.00, 2.50)
*4th*	1.92^b^ (1.69, 2.20)	1.94^b^ (1.67, 2.25)	1.80^b^ (1.63, 2.00)
*Upper 20% (ref)*	1.00	1.00	1.00

^a ^5% level of significance

^b^ 1% level of significance

The presence of a chronic patient in the household increases the risk of CHE by 1.17 times (AOR = 1.17, 95% CI = 1.05, 1.31) at 10% of the THCE threshold. The probability of facing CHE from hospitalization at private facilities, as compared to public facilities, increases 3.03 and 2.28 times at 10% of THCE and at 25% of NFE respectively. Similarly, the likelihood of CHE due to non-communicable diseases are 64% and 52% higher than communicable diseases. Moreover, the chances of CHE were 2.78, 2.49, 2.01, and 1.92 times higher for the lowest 20%, 2^nd^, 3^rd^, and 4^th^ quintiles compared with the upper 20% of households at 10% of THCE threshold.

Statistically significant determinants of distress health financing are the number of earning members, number of chronic patients, number of members hospitalized in the last 12 months, the type of hospital provider, diseases type, administrative division, place of residence, and wealth quintiles. Similar to CHE, households with one and two or more chronic patients were more likely to expect distress financing by 1.40 and 1.51 times, respectively. In addition, obtaining healthcare from private hospitals and due to non-communicable diseases increases the likelihood of distress financing by 1.37 and 1.39 times respectively. The likelihoods of experiencing distress health financing were 3.56, 2.11, 2.23 and 1.80 times higher for lowest four quintiles respectively.

## Discussion

This study was designed to provide disaggregated incidences of hospitalization, and estimates of OOPE, CHE, and distress financing by type of diseases and healthcare providers. In addition, our research also aims to investigate inequalities in access to hospital care and the impact of OOPE on hospitalization. Several major findings from the nationally representative survey data are revealed in this study. The latest nationwide data observed that NCDs had a greater rate of hospitalization and financial hardship than communicable diseases in Bangladesh. We also found that private hospitals had a higher financial burden on hospitalization than public hospitals. Furthermore, we observed that poor vulnerable households are suffering more from the CHE and distress financing, although they utilized less inpatient (hospitalization) healthcare services than rich households.

The overall incidence rate of hospitalization in our study was 175 per 10,000 population, with higher hospitalization rates due to NCDs rather than communicable diseases. A study also reported higher self-reported incidence of NCDs in rural Bangladesh than communicable diseases [8] and hospitalizations at the district level hospitals were also found to be comparatively higher for NCDs than communicable diseases [27]. Similarly, a study in India (a neighboring country) also found that hospitalization rates for NCDs and injuries are slightly higher in India than communicable diseases [25]. Our study also found that hospitalization rates in Bangladesh were greater in public health facilities than in private facilities, indicating that public health facilities are still Bangladesh’s core health service providers on hospitalization, despite the presence of a large private sector. Our findings, in this regard, were comparable in Nepal, Myanmar, Sri Lanka, and the Philippines, revealing that public facilities are more prevalent for inpatient care [17]. On the other hand, hospitalization in private facilities was found to be significantly higher in India and Pakistan [17].

Not unexpectedly, our study observed that the average OOPE on hospitalization was nearly twice as high in private facilities as it was in public facilities. Our finding on a higher OOPE in private facilities for hospitalization was aligned to those reported in India, Pakistan, Nepal, Sri Lanka, and Myanmar [17]. We also found that, NCDs had 1.5 times higher OOPE than communicable diseases, particularly with cancer having the highest average OOPE, followed by liver and heart diseases. A similar pattern has also been reported in India, Pakistan, Nepal, and Sri-Lanka where the burden of OOPE are significantly higher among households with NCDs [28]. A study based on rural areas in Bangladesh also found a higher intensity of OOPE among NCD patients who were treated from micro-health insurance scheme operated hospitals, particularly for cancer [8]. Similar findings were also reported by another study based on a metropolitan city in Bangladesh, implying that liver and heart diseases are the leading causes of OOPE [9].

In our study, the estimated incidence of CHE was 26.1% (at 10% of THCE) and 21.5% (at 25% of NFE) on hospitalization. This indicates an increasing burden of CHE in Bangladesh as earlier studies estimated incidences of CHE from the same survey of 24.6% in 2016 and 14.2% in 2010 (at 10% of THCE) by assessing both hospitalization and outdoor services [3, 6]. Furthermore, our research found that the incidence of CHE was much greater among patients who received care from private facilities, and who were predominantly treated for NCDs. More precisely, we discovered that cancer had the greatest devastating impact, followed by liver diseases and paralysis. These findings are consistent with studies conducted in rural Bangladesh, India, China, Vietnam, Pakistan, Nepal, and Sri-Lanka highlighting that higher CHE for NCDs in general, with cancer and liver diseases in particular, are associated with higher CHE [8, 25, 28, 29].

However, according to our study, more than half of the hospitalized cases in Bangladesh had distress financing due to financial crisis. Similar to other estimated indicators in this study, distress financing is also higher among those who received care from private facilities, and for NCDs such as mental health, cancer, liver diseases, and paralysis. In addition, we observed that one-third of households with at least one hospitalization had both CHE and distress financing. Liver diseases had the highest level of incidence for both CHE and distress financing, followed by female diseases, cancer, paralysis, kidney diseases, and injury/road accident. A study in a metropolitan city (Rajshahi) in Bangladesh found that hospitalization caused 37% of distress financing [10]. Another study of 40 LMICs found an average of 32% distress financing when both hospitalization and outdoor services were considered [30]. However, the definition of distress financing in those studies does not include aid from friends and relatives, but instead just borrowing and selling assets.

More hospitalization in public facilities could be due to subsidized healthcare, availability of eminent health specialists, and emergency patient management [31], regardless of the fact that perceived quality of service and patient satisfaction are greater in private facilities than public facilities [32, 33]. The momentous financial burden of hospitalization in Bangladesh signifies the dependency on OOPE in both public and private facilities, as OOPE in total health expenditure grew from 60 to 67% from 2010 to 2015, while government spending declined from 26 to 23% [4]. The fact that financial hardship was higher in private health facilities than in public facilities could be attributable to high treatment costs at private facilities as they offer more personalized and quality healthcare with the preference of physicians and modern technologies without having any financial support from the government. Long hospital stays for NCDs particularly for cancer, liver disease, and heart disease combined with high medical treatment costs, as well as indirect costs such as diet, lodging, informal payments, and transportation, result in higher total OOPE that places a greater financial strain on households.

Our research found that having more children under the age of five in a household increases the risk of CHE on hospitalization in Bangladesh. Previous studies also found that having children in the household increases the likelihood of encountering CHE in both inpatients and outdoor health services in Bangladesh [10, 24]. This could be due to the fact that children are more susceptible to frequent health hazards, such as diarrheal infections, fever, and respiratory illnesses like pneumonia, which demand regular hospital stays, ultimately resulting in higher OOPE for households [34, 35]. Similarly, having chronic patients in the household was also linked to a higher risk of CHE and distress financing, as chronic patients had longer hospital stays and required regular medication and follow-up for longer periods of time. A study in Bangladesh also came to the same conclusion: chronic care can put a household’s finances under constant hardship, which might lead to an increase in OOPE, CHE, and may demand distress financing [24]. Larger households, on the other hand, appear to have a lower incidence of CHE than smaller households. This finding could be related with number of earning members; large households may have more earning members (which significantly reduces the risk of CHE and distress financing), as well as more opportunities to have a higher income in total and savings to meet hospitalization expenses. A recent study in Bangladesh also found that large households are less likely to suffer from CHE [3]. A longitudinal study in India, on the other hand, demonstrated that large households are more likely to have CHE than small households [36].

Our research highlights that UHC financial protection indicators are disproportionately concentrated in poor households, implying that poor households are more prone to experience CHE and distress financing during hospitalization than rich households. However, less access to hospital care for poor households indicate the presence of access barriers due to their financial inability. The health spending of rich households has been associated to their higher ability to pay and the quality of treatment they received, as they faced less financial hardship due to hospitalization. On the other hand, for poor households a small amount of health care spending can push them to CHE, for which they finance by borrowing, selling assets, or seeking assistance from friends or family. Indeed, they may not even seek healthcare at all since they do not have sufficient money to meet healthcare costs.

The extent and severity of financial hardship associated with OOPE on hospitalization, as investigated in this study, strongly implies the need for national-level social health security schemes with a comprehensive benefit package to cover all citizens, with a particular focus on the poor. Although putting such a national plan in place is arduous; pro-poor, employment-based, and community-based or micro-health insurance schemes could all eventually lead towards a national social health security system in Bangladesh. More importantly, the government should take the initiative to engage the private sector including NGOs and also need to provide sufficient financial and technical support to them, in establishing a comprehensive social health security scheme to ensure better health access for the population and to achieve national goals. Higher financial burdens in the private sector in Bangladesh also bring policymakers’ attention to the need to enact regulatory measures for the private sector. At the same time, regular monitoring should be implemented in both public and private facilities to ensure certain quality standards. Specialized healthcare services, particularly those based on new healthcare technologies, which are currently unavailable at the public facilities should be introduced to protect poor households from high OOPE that occurs due to seeking healthcare from private facilities. Furthermore, subsidized programmes targeting diseases with significant treatment costs, such as cancer, heart disease, liver disease, and kidney disease, should be developed to address the growing financial burden of NCDs. At the same time, this study suggests that treatment for those high-cost diseases be incorporated into existing health insurance benefit packages. Finally, in order to reduce reliance on OOPE, the government should consider increasing its allocations to the health sector, since public health expenditure in Bangladesh is significantly lower than other South Asian countries.

## Study limitations

Our analysis was initially limited to inpatient i.e., hospitalization care, which may have underestimated the financial burden of OOPE, CHE, and distress financing. Since HIES has collected healthcare expenditure data for 365 days of inpatient (hospitalization) and 30 days of outpatient services prior to the survey periods, we had two options for making expenditure data uniform: converting either 30 days expenditure to 365 days or 365 days expenditure to 30 days. However, such a conversion may underestimate/overestimate the actual burden of OOPE [3]. Since hospitalization is one of the leading reasons of CHE and distress financing, we chose to confine our focus to hospitalization. Second, we use THCE and NFE as proxy measures of income to estimate CHE; however, in some situations, this may exaggerate CHE for wealthy households whose income is higher than THCE. Third, recall bias is expected to be strong in the 365 days leading up to the survey, which could affect our estimation if participants provide misinformation due to the need to recall information over a long period of time. Finally, another limitation is the fact that our analysis is based on cross-sectional data rather than longitudinal data. Despite these limitations, according to the authors’ knowledge, this study is the first in Bangladesh to provide disease-specific incidence of distress financing and catastrophic OOPE on hospitalization by public and private facilities.

## Conclusion

Our research revealed substantial financial burden in terms of CHE and distress financing due to OOPE on hospitalization. We also found that private facilities had higher OOPE, CHE, and distress financing than public facilities. In Bangladesh, NCDs had a greater financial hardship than communicable diseases when it came to hospitalization. Cancer, heart disease, liver disease, paralysis, accident/injury, and kidney disease, in particular, are causing increased financial hardship. Finally, destitute households are more likely than wealthy households to suffer from CHE and distress financing. These findings present potential barriers to Bangladesh’s goal of reaching UHC, and highlight the need for a national social health protection scheme, and to reform current healthcare services in public facilities according to disease burden. This study intended to assist policymakers in Bangladesh in taking the required steps to safeguard households from financial hardship and to prioritize needs in public facilities. Our research suggests that in order to reduce CHE and distress financing, a mixture of alternative healthcare financing channels apart from OOPE should be investigated, as the current financing method has struggled to provide financial protection to lower income households and is insufficient to ensure UHC.

## Data Availability

The HIES 2016 dataset is publicly available upon request by the Bangladesh Bureau of Statistic (BBS) to use this dataset.

## References

[1] UN General Assembly (2015). Transforming our world: the 2030 agenda for sustainable development.

[2] World Health Organization. Regional Office for South-East Asia. Monitoring progress on universal health coverage and the health-related Sustainable Development Goals in the WHO South-East Asia Region. World Health Organization. Regional Office for South-East Asia; 2019.

[3] Ahmed S, Ahmed MW, Hasan MZ, Mehdi GG, Islam Z, Rehnberg C, et al. Assessing the incidence of catastrophic health expenditure and impoverishment from out-of-pocket payments and their determinants in Bangladesh: evidence from the nationwide Household Income and Expenditure Survey. Int Health. 2016;2021:1–13. 10.21203/RS.3.RS-325300/V1. 10.1093/inthealth/ihab015 PMC8769950 33823538

[4] Ministry of Health and Family Welfare. Bangladesh Health Accounts 1997–2015. Health Economics Unit, Health Services Division, Ministry of Health and Family Welfare, GoB. Dhaka, Bangladesh; 2018.

[5] Xu K, Evans DB, Carrin G, Aguilar-Rivera AM, Musgrove P, Evans T (2007). Protecting households from catastrophic health spending. Health Aff.

[6] Khan JAM, Ahmed S, Evans TG (2017). Catastrophic healthcare expenditure and poverty related to out-of-pocket payments for healthcare in Bangladesh- An estimation of financial risk protection of universal health coverage. Health Policy Plan.

[7] Sarker AR, Sultana M, Alam K, Ali N, Sheikh N, Akram R (2021). Households’ out-of-pocket expenditure for healthcare in Bangladesh: A health financing incidence analysis. Int J Health Plann Manage.

[8] Hamid SA, Ahsan SM, Begum A (2014). Disease-specific impoverishment impact of out-of-pocket payments for health care: evidence from rural Bangladesh. Appl Health Econ Health Policy.

[9] Rahman MM, Zhang C, Swe KT, Rahman MS, Islam MR, Kamrujjaman M (2020). Disease-specific out-of-pocket healthcare expenditure in urban Bangladesh: a Bayesian analysis. PLoS One.

[10] Islam MR, Rahman MS, Islam Z, Nurs CZB, Sultana P, Rahman MM (2017). Inequalities in financial risk protection in Bangladesh: an assessment of universal health coverage. Int J Equity Health.

[11] Boutayeb A. The Burden of Communicable and Non-Communicable Diseases in Developing Countries. Handb Dis Burdens Qual Life Meas. 2010. p. 531–546.

[12] World Health Organization (2018). Noncommunicable diseases country profiles 2018.

[13] Noor R, Munna S (2015). Emerging diseases in Bangladesh: current microbiological research perspective. Tzu Chi Med J.

[14] Mejia N, Pallas SW, Saha S, Udin J, Ishtiaque Sayeed KM, Garrett DO (2020). Typhoid and Paratyphoid Cost of Illness in Bangladesh: patient and health facility costs from the surveillance for enteric fever in Asia project II. Clin Infect Dis.

[15] Sultana M, Alam NH, Ali N, Faruque ASG, Fuchs GJ, Gyr N (2021). Household economic burden of childhood severe pneumonia in Bangladesh: a cost-of-illness study. Arch Dis Child.

[16] Sarker A, Sultana M, Mahumud R, Khan J, Meer R, Morton A (2017). Economic burden of hospitalized diarrheal disease in Bangladesh. Value Heal.

[17] Saksena P, Xu K, Elovainio R, Perrot J (2012). Utilization and expenditure at public and private facilities in 39 low-income countries. Trop Med Int Heal.

[18] Joarder T, Chaudhury TZ, Mannan I (2019). Universal health coverage in Bangladesh: activities, challenges, and suggestions. Adv Public Heal.

[19] Ahmed S, Hasan MZ, Ahmed MW, Dorin F, Sultana M, Islam Z (2018). Evaluating the implementation related challenges of Shasthyo Suroksha Karmasuchi (health protection scheme) of the government of Bangladesh: a study protocol. BMC Health Serv Res.

[20] Bangladesh Bureau of Statistics. Household Income Expenditure Survey. Dhaka, Bangladesh; 2016. Available from: http://dx.doi.org/10.1016/j.jsames.2011.03.003%0Ahttps://doi.org/10.1016/j.gr.2017.08.001%0Ahttp://dx.doi.org/10.1016/j.precamres.2014.12.018%0Ahttp://dx.doi.org/10.1016/j.precamres.2011.08.005%0Ahttp://dx.doi.org/10.1080/00206814.2014.902757%0Ahttp://dx

[21] Pan American Health Organization and World Health Organization. Non-communicable Diseases. Top. Dis. 2022. Available from: https://www.paho.org/en/topics/noncommunicable-diseases. Cited 2021 Jun 17.

[22] Xu K, Evans DB, Kawabata K, Zeramdini R, Klavus J, Murray CJL (2003). Household catastrophic health expenditure: a multicountry analysis. Lancet.

[23] Wagstaff A, van Doorslaer E (2003). Catastrophe and impoverishment in paying for health care: with applications to Vietnam 1993–1998. Health Econ.

[24] Rahman MM, Gilmour S, Saito E, Sultana P, Shibuya K (2013). Health-related financial catastrophe, inequality and chronic illness in Bangladesh. PLoS One.

[25] Kastor A, Mohanty SK (2018). Disease-specific out-of-pocket and catastrophic health expenditure on hospitalization in India: do Indian households face distress health financing?. PLoS One.

[26] Ir P, Jacobs B, Asante AD, Liverani M, Jan S, Chhim S (2019). Exploring the determinants of distress health financing in Cambodia. Health Policy Plan.

[27] Rahman MM, Kabir MA, Mehjabin M (2019). Pattern of non-communicable diseases among the admitted patients in a District Level Hospital of Bangladesh. Bangladesh Hear J.

[28] Rijal A, Adhikari TB, Khan JAM, Berg-Beckhoff G (2019). The economic impact of noncommunicable diseases among households in South Asia and their coping strategy: a systematic review. PLoS One.

[29] Saksena P, Evans D, Xu K (2011). Impact of out-of-pocket payments for treatment of non-communicable diseases in developing countries: a review of literature.

[30] Kruk ME, Goldmann E, Galea S (2009). Borrowing and selling to pay for health care in low- and middle-income countries. Health Aff.

[31] Miah MS, Naser A, Saif M, Afrin ST (2020). A comparative analysis of quality of health care between public and private hospitals in Bangladesh. BUFT J Bus Econ.

[32] Andaleeb SS (2000). Public and private hospitals in Bangladesh: Service quality and predictors of hospital choice. Health Policy Plan.

[33] Begum F, Said J, Hossain SZ, Hasan MJ, Binti N. Healthcare Cost and Patient Satisfaction: A Comparative Analysis Between Public and Private Hospitals in Bangladesh. Res Sq. 2019;1–19.

[34] Sarker AR, Sultana M, Mahumud RA, Sheikh N, Van Der Meer R, Morton A (2016). Prevalence and health care-seeking behavior for childhood diarrheal disease in Bangladesh. Glob Pediatr Heal.

[35] Sultana M, Sarker AR, Sheikh N, Akram R, Ali N, Mahumud RA (2019). Prevalence, determinants and health care-seeking behavior of childhood acute respiratory tract infections in Bangladesh. PLoS One.

[36] Swetha NB, Shobha S, Sriram S (2020). Prevalence of catastrophic health expenditure and its associated factors, due to out-of-pocket health care expenses among households with and without chronic illness in Bangalore, India: a longitudinal study. J Prev Med Hyg.

